# Examining differences in suicidality between and within mental health disorders and sexual identity among adults in the United States

**DOI:** 10.3934/publichealth.2021051

**Published:** 2021-09-18

**Authors:** David Adzrago, Ikponmwosa Osaghae, Nnenna Ananaba, Sylvia Ayieko, Pierre Fwelo, Nnabuchi Anikpezie, Donna Cherry

**Affiliations:** 1 Center for Health Promotion and Prevention Research, The University of Texas Health Science Center at Houston, Houston, Texas, USA; 2 Department of Epidemiology, Human Genetics & Environmental Sciences, The University of Texas Health Science Center at Houston School, Houston, Texas, USA; 3 Department of Population Health Science, The University of Mississippi Medical Center, Jackson, Mississippi, USA; 4 Department of Social Work, East Tennessee State University, Johnson City, Tennessee, USA

**Keywords:** suicidality, sexual identity, mental health disorder, substance use disorder

## Abstract

**Background:**

Suicide is a leading but preventable cause of death and is preceded by domains of thoughts, plans, and attempts. We assessed the prevalence of suicidality domains and determined the association of suicidality domains with sexual identity, mental health disorder symptoms, and sociodemographic characteristics.

**Methods:**

We used the 2019 National Survey on Drug Use and Health (NSDUH) data to perform weighted multivariable logistic regression and margins analyses to examine between and within-group differences in suicidality by sexual identity among adults aged ≥ 18 years.

**Results:**

About 4.89%, 1.37%, and 0.56% of the population experienced suicidal thoughts, plans, and attempts, respectively. Those aged 18–25 years old had a higher odds of suicidality compared to those aged 26 years or older. Compared to those who reported having no alcohol use dependence, illicit drug use dependence, and major depressive episodes (MDEs), those who reported alcohol use dependence, illicit drug use dependence, and MDE had higher odds of suicidal thoughts, plans, and attempts. Between all sexual identity groups, bisexuals who experienced MDEs had the highest probability of having suicidal thoughts while lesbians and gays who experienced MDE showed a higher probability of suicidal plans and attempts compared to heterosexuals. Within each sexual identity group, the probability of having suicidal thoughts, suicidal plans, and suicidal attempts was higher for those who had experienced MDEs compared to those who had not experienced MDEs.

**Conclusion:**

Substance use disorder and MDE symptoms were associated with increased suicidality, especially among young adults and sexual minority people. This disparity underscores the need for tailored interventions and policies to enhance the provision of prompt mental health screening, diagnosis, and linkage to care for mental health services, particularly among the most vulnerable in the population.

## 1. Introduction

Over the years, the global burden of suicide has been on the rise. Between 1990 and 2016, the total number and rates of deaths by suicide significantly increased by 6.7% and 33%, respectively [1],[2]. In the United States (US), suicide is the second leading cause of death among people between the ages of 15–34 years and the 10th leading cause of death across all age groups [3]. While the trends in suicide mortalities are alarming, studies indicate that for each death by suicide, there are approximately 30 suicide attempts reported [4]. Suicidal ideations and plans are also risk factors for future suicidal behavior [5]. In 2019, the Substance Abuse and Mental Health Service Administration (SAMHSA) estimated that 12 million people in the US had suicidal ideations, 3.8 million individuals had suicidal plans, and 1.4 million adults attempted suicide [6]. The overall prevalence of suicidal thoughts among adults in the US is approximately 4.8% and is 8-times higher than suicidal attempts [7].

Suicidality is a spectrum that encompasses suicidal ideations, suicidal plans, and actual suicide attempts [8]. These suicidal behaviors are risk factors for completed suicide and have devastating effects on individuals. People who unsuccessfully attempt suicide may also sustain serious injuries with long-term effects on their health [9]. Suicidality may be closely associated with mental health disorder symptoms, including symptoms of depressive, mood, and substance use disorders [10]. Research suggests that depression, hopelessness, and most mental health disorder symptoms may be associated with suicidal ideations [11],[12]. Some scholars argue that since substance abuse is a comorbid condition with depression, it is indirectly associated with suicidal thoughts among high-risk populations [13],[14]. Excessive use of alcohol and drugs is also linked with higher levels of suicidal ideation [15],[16].

Sexual identity (i.e., heterosexual, lesbian, gay, and bisexual) may be associated with a significant risk of mental health disorder symptoms [17],[18]. Research suggests that people who identify as lesbian, gay, and bisexual (LGB), compared to those who identify as heterosexual, have higher risks of suicidality because they are more exposed to stigma, discrimination, and victimization [19]. However, the evidence of differences in suicidality risks between LGB people and heterosexual people is not conclusive [20]–[24]. In a meta-analysis that examined mental health in LGB persons, sexual minority people were found to have a higher risk of suicidality than heterosexual people [23]. However, the association did not vary across sexual orientation categories (i.e., heterosexual, lesbian, gay, and bisexual). A limitation to this study is that it has analyzed suicidal attempts as a single variable to defined suicidality, although suicidality comprises ideation, plan, and attempt. Additionally, some of the sample sizes of the studies included in the meta-analysis were as low as 104 participants. In another systematic review and meta-analysis, the authors found that the association between sexual orientation and suicide was established in only a few studies [24]. It was further determined that only a few studies examined factors associated with suicide attempts or suicide among LGB populations. It is unclear if there are any differences in suicidality between and within sexual identity subgroups. To provide effective interventions to reduce suicidality and the effects of suicidal behaviors, it is critical to understand how these variables interact and how they impact sexual minorities particularly. It is also essential to use a large national sample to provide nationally representative estimates that would inform large public suicidality-related interventions.

This study examines the associations of mental health disorder symptoms and sexual identity with suicidality. We used the 2019 National Survey on Drug Use and Health (NSDUH) data, a nationally representative survey, to bridge the gap and expand on the limited literature on suicidality and its risk factors between and within sexual minorities and heterosexual people in the United States. The main aims of our analysis are to: (1) assess the prevalence of suicidality domains among sexual identity subgroups; (2) determine the association between suicidality domains versus sexual identity, mental health disorder symptoms, and sociodemographic variables; and (3) evaluate differences in suicidality domains between and within mental health disorder symptoms and sexual identity groups.

## 2. Methods

### 2.1. Study design

We used the 2019 National Survey on Drug Use and Health (NSDUH) public-use data for this study. The NSDUH is an annual survey of the US civilian, noninstitutionalized population aged 12 years or older, sponsored by the SAMHSA, US Department of Health and Human Services. This state-based, multistage area probability sample survey collects information on suicidality, use of tobacco, alcohol, prescription psychotherapeutic drugs, and other substances. A detailed description of the survey design and methodology is available in the Center for Behavioral Health Statistics and Quality [25]. The 2019 NSDUH was the most recent survey year available at the time of this study. The public-use data file included information for 56,136 individuals; however, our study is limited to adults aged 18 years and older (N = 42739). The data is de-identified and does not contain individual or state identifiers. Therefore, a review from an Institutional Review Board was not required.

### 2.2. Measures

#### 2.2.1. Dependent variables

Suicidal thoughts, plans, and attempts: Three questions in the survey assessed suicidal thoughts, suicidal plans, and suicidal attempts in the past year. The participants were asked whether they had seriously thought about committing suicide in the past year, whether they had made plans to commit suicide in the past year, and whether they had attempted to commit suicide in the past year. Each of these variables was a dichotomous variable (yes/no). These variables in the survey were treated separately because they were not mutually exclusive (i.e., some participants could experience one, two, or all suicidality domains). Hence, we analyzed them as individual outcome variables.

#### 2.2.2. Independent variables

Major depressive episode (MDE) symptoms: Depression symptom variables in the survey were based on the Major depressive episode (MDE) as defined by the Diagnostic and Statistical Manual of Mental Disorders 5th edition (DSM-5) [26],[27]. This variable was dichotomized (yes/no) in NSDUH to indicate whether the participants had likely experienced an MDE in the past year or not (see details in Center for Behavioral Health Statistics and Quality [25],[28]). Participants who had a lifetime MDE (i.e., if they had at least five of nine attributes of MDE described in NSDUH codebook) and felt depressed or lost interest or pleasure in daily activities for 2 weeks or longer in the past 12 months, while having some other lifetime MDE symptoms, were classified as having MDE symptoms in the past year [28]. Those who had no lifetime MDE (i.e., if they had few than five of nine attributes of MDE) or had lifetime MDE symptoms but no period of depression lasting 2 weeks or longer in the past 12 months while having other symptoms for lifetime MDE, were classified as not having MDE symptoms in the past year [28].

Alcohol use dependence: Alcohol use dependence in the past year was measured in NSDUH based on DSM-4 criteria [6],[29]. Participants who, based on self-report, met at least three of the seven alcohol use dependence criteria, were classified as having experienced alcohol use dependence in the past year. Otherwise, they were classified as not having experienced alcohol use dependence in the past year. These criteria include: (1) spent a great deal of time over a month or more getting, using or getting over the effects of alcohol; (2) used alcohol more often than intended or was unable to keep set limits on alcohol use; (3) needed to use alcohol more than before to get desired effects or noticed that same amount of alcohol use had less effect than before; (4) inability to cut down or stop using alcohol every time tried or wanted to; (5) continued to use alcohol even though it was causing problems with emotions, nerves, mental health, or physical problems; (6) alcohol use reduced or eliminated involvement or participation in important activities; and (7) reported experiencing two or more alcohol withdrawal symptoms at the same time that lasted longer than a day after alcohol use was cut back or stopped.

Illicit drug use dependence: Illicit drug use dependence in the past year was measured based on DSM-4 criteria [6],[29]. Participants who reported to be dependent on any illicit drugs (i.e., marijuana, hallucinogens, inhalants, methamphetamine, tranquilizers, cocaine, heroin, pain relievers, stimulants, or sedatives) were classified as having illicit drug use dependence in the past year. Participants who met three or more of the six dependence criteria were classified as having experienced illicit drug use dependence in the past year. Otherwise, they were classified as not dependent on illicit drug use in the past year. The six criteria include: (1) spent a great deal of time over a month getting, using, or getting over the effects of the substance; (2) unable to keep set limits on substance use or used more often than intended; (3) needed to use substance more than before to get desired effects or noticed that using the same amount had less effect than before; (4) unable to cut down or stop using the substance every time he or she tried or wanted to; (5) continued to use substance even though it was causing problems with emotions, nerves, mental health, or physical problems; and (6) reduced or gave up participation in important activities due to substance use. An additional criterion was included for a pain reliever, cocaine, heroin, sedative, stimulant, or methamphetamine dependence. This 7th criterion involves experiencing substance-specific withdrawal symptoms at one time that lasted for longer than a day after cutting back or stopped using.

Sociodemographic characteristics: The following sociodemographic characteristics were assessed based on findings from previous studies [4],[18]–[24]: age (18–25, 26–34, 35–49, 50–64, 65 ≥), gender (male/female), sexual identity (heterosexual, lesbian or gay, and bisexual), race/ethnicity (non-Hispanic White, non-Hispanic Black/African American, Hispanic, and Other race [non-Hispanic Native American/Alaskan Native, non-Hispanic Asian American, non-Hispanic Native Hawaiian/Other Pacific Islander, and non-Hispanic more than one race]), level of education completed (Twelfth grade or less grade, High School diploma/GED, some college credit but no degree, Associate's degree, and college graduate or higher), employment status (employed full time, employed part-time, and unemployed), and total family income (< $20,000; $20,000 to $49,999; $50,000–$74,999; and ≥ $75,000). We analyzed these variables as categorical variables in this paper.

### 2.3. Statistical analysis

Descriptive analysis was performed to describe the weighted percentages and frequencies of the participants' sociodemographic characteristics and the prevalence of mental health disorder symptoms by suicidal thoughts, suicidal plans, and suicidal attempts (see Table 1). The weighted frequencies and percentages were presented in Table 1 by columns and should be interpreted by column frequencies and percentages. Bivariate analyses were conducted to determine the association between the outcome variables (suicidal thoughts, plans, and attempts) and sociodemographic characteristics and mental health disorder symptoms using chi-square tests (see Table 1). The variables that were significantly associated with the outcomes at the bivariate level (p < 0.05) were entered into the weighted multivariate logistic regression model (see Table 2). Three multivariable logistic regression models were conducted to examine associations. In model 1, we examined suicidal thoughts as the outcome variable; in model 2, we analyzed suicidal plans as the outcome variable; and in model 3 we used suicidal attempts as the outcome variable. In each model, we assessed the association between the outcome variable and mental health disorder risks and sexual identity, adjusting for sociodemographic variables. Age, gender, sexual identity, race/ethnicity, level of education completed, employment status, total family income, alcohol use dependence, illicit drug use dependence, and MDE symptoms were included in models 1 and 3. Model 2 included similar sociodemographic characteristics excluding race/ethnicity because it was not significant at the bivariate level. After examining each model, we further examined the marginal probabilities of MDE symptoms and sexual identity interactions on each suicidality domain. We performed the marginal analyses using marginal estimates and marginsplot to determine between and within-group differences in each suicidality domain by the MDE symptoms and sexual identity. Thus, marginal analyses help to further probe interaction effects on an outcome or dependent variable by estimating the predicted or expected values, which provide between and within-group effects on an outcome [30]. We also examined the interaction between gender and sexual identity on each suicidality domain, but this was not statistically significant (p > 0.05), so we did not proceed to perform the marginal analysis.

To achieve nationally representative estimates and interpretations, our statistical analyses were weighted using the NSDUH survey weight. The survey weight accounts for the weighting and clustering effects, including unequal probabilities of sampling, non-response, and post-stratification adjustments. To obtain accurate variance estimates, we also used the NSDUH nesting variables to capture explicit stratification and to ascertain clustering with the data [25]. All the analyses were conducted at an alpha level of 0.05. The data were analyzed with STATA/SE, version 16.1 [31].

## 3. Results

### 3.1. Descriptive and bivariate statistics

The descriptive and bivariate statistics of suicidality among US adults are presented in Table 1. We presented the results for past-year suicidal thoughts, plans, and attempts, respectively. The majority of the adult population was aged 35–49 (24.37%) and 50–64 years (24.99%), females (51.77%), heterosexual (94.07%), non-Hispanic White (63.19%), completed college or higher education (33.12%), employed full time (49.67%), and had a total family income of $75,000 or more (40.97%). Significant proportions of the participants experienced past-year alcohol use dependence (3.14%), illicit drug use dependence (2.18%), and MDE symptoms (7.89%).

Suicidal thoughts: About 4.89% of the population had suicidal thoughts. The bivariate analysis indicated that age, gender, sexual identity, race/ethnicity, level of education completed, employment status, total family income, experience with alcohol use dependence, illicit drug use dependence, and MDE symptoms were associated with suicidal thoughts in the past year. The majority of the population who had suicidal thoughts was aged 18–25 (33.34%), females (55.48%), had some college credit but no degree (29.7%), had full-time employment (45.87%), and had a total family income of $20,000–$49,999 (31.77%). Among those with suicidal thoughts, significant proportions were bisexuals (15.96%) and Hispanics (17.07%). The population who had suicidal thoughts had experiences with alcohol use dependence (12.12%), illicit drug use dependence (11.18%), and MDE (51.6%).

Suicidal plans: The prevalence of suicidal plans was 1.37%. The same percentage of the population who responded to the suicidal thought survey also completed the suicidal plan survey. From the bivariate analysis, only the race/ethnicity of the population was not significantly associated with past-year suicidal plans (p = 0.870). The proportions of the population who had past-year suicidal plans were within ages 18–25 years (38.98%), females (59.46%), had some college credit but no degree (30.74%), had full-time employment (39.98%), and total family income of $20,000–$49,999 (30.52%). They also had experiences with alcohol use dependence (14.43%), illicit drug use dependence (15.82%), and MDE (51.6%).

Suicidal attempts: About 0.56% of the population had suicidal attempts. Age, gender, sexual identity, race/ethnicity, level of education completed, employment status, total family income, experience with alcohol use dependence, illicit drug use dependence, and MDE symptoms were associated with past-year suicidal attempts. A significant proportion of the population who reported to have had past-year suicidal attempts was aged 18–25 years (44.27%), females (62.05%), had some college credit but no degree (30.08%), had full-time employment (36.47%), and total family income of $20,000–$49,999 (33.1%). The population who reported experiences with alcohol use dependence (15.19%), illicit drug use dependence (18.03%), and MDE symptoms (51.6%), reported suicidal attempts in the past year.

**Table 1.** Suicidality among US adults in 2019 using weighted surveys, by demographic characteristics, substance use, and mental health disorder symptoms.

		No Suicidal Thoughts	Suicidal Thoughts			No Suicidal Plans	Suicidal Plans			No Suicidal Attempts	Suicidal Attempts	
	Overall N (%)	n (%)	n (%)		Overall N (%)	n (%)	n (%)		Overall N (%)	n (%)	n (%)	
Variable	248539359 (100%)	236386858 (95.11%)	12152500 (4.89%)	*p*-value	248538454 (100%)	245142573 (98.63%)	3395881 (1.37%)	*p*-value	248516493 (100%)	247118941 (99.44%)	1397552 (0.56%)	*p*-value
Sociodemographics												
Age:				<0.001				<0.001				<0.001
18–25	33339839 (13.41)	29288008 (12.39)	4051831 (33.34)		33338934 (13.41)	32015227 (13.06)	1323707 (38.98)		33329940 (13.41)	32711254 (13.24)	618685 (44.27)	
26–34	40057659 (16.12)	37359510 (15.8)	2698149 (22.2)		40057659 (16.12)	39308143 (16.03)	749515 (22.07)		40057659 (16.12)	39744055 (16.08)	313604 (22.44)	
35–49	60555602 (24.37)	57964742 (24.52)	2590859 (21.32)		60555602 (24.37)	59839114 (24.41)	716487 (21.1)		60555602 (24.37)	60275909 (24.39)	279692 (20.01)	
50–64	62116348 (24.99)	60343847 (25.53)	1772501 (14.59)		62116348 (24.99)	61722943 (25.18)	393405 (11.58)		62103382 (24.99)	62002986 (25.09)	100396 (7.18)	
65 or Older	52469912 (21.11)	51430751 (21.76)	1039161 (8.55)		52469912 (21.11)	52257145 (21.32)	212766 (6.27)		52469912 (21.11)	52384737 (21.2)	85174 (6.10)	
Gender:				0.006				0.003				0.014
Male	119875576 (48.23)	114464931 (48.42)	5410645 (44.52)		119875576 (48.23)	118498774 (48.34)	1376802 (40.54)		119875576 (48.24)	119345161 (48.29)	530415 (37.95)	
Female	128663783 (51.77)	121921928 (51.58)	6741855 (55.48)		128662878 (51.77)	126643799 (51.66)	2019079 (59.46)		128640917 (51.76)	127773781 (51.71)	867137 (62.05)	
Sexual identity:				<0.001				<0.001				<0.001
Heterosexual	229561755 (94.07)	219992206 (94.82)	9569549 (79.74)		229561755 (94.07)	227206566 (94.41)	2355188 (69.99)		229545437 (94.08)	228581910 (94.22)	963527 (69.41)	
Lesbian or Gay	4855550 (1.99)	4339652 (1.87)	515899 (4.30)		4855550 (1.99)	4607572 (1.92)	247978 (7.37)		4855550 (1.99)	4761450 (1.96)	94101 (6.78)	
Bisexual	9606514 (3.94)	7690633 (3.32)	1915881 (15.96)		9605609 (3.94)	8843722 (3.68)	761888 (22.64)		9599966 (3.93)	9269331 (3.82)	330636 (23.82)	
Race/Ethnicity:				0.027				0.870				0.033
Non-Hispanic White	157061528 (63.19)	149075875 (63.06)	7985653 (65.71)		157060623 (63.19)	154909592 (63.19)	2151031 (63.34)		157043619 (63.19)	156286140 (63.24)	757479 (54.2)	
Non-Hispanic Black/African American	29574267 (11.9)	28415169 (12.02)	1159098 (9.54)		29574267 (11.9)	29205087 (11.91)	369180 (10.87)		29569310 (11.9)	29329926 (11.87)	239384 (17.13)	
Sociodemographics												
Hispanic	40825268 (16.43)	38751307 (16.39)	2073960 (17.07)		40825268 (16.43)	40238164 (16.41)	587104 (17.29)		40825268 (16.43)	40562106 (16.41)	263162 (18.83)	
Other race	21078296 (8.48)	20144508 (8.52)	933788 (7.68)		21078296 (8.48)	20789730 (8.48)	288566 (8.50)		21078296 (8.48)	20940769 (8.47)	137527 (9.84)	
Level of education completed:				<0.001				<0.001				<0.001
Twelfth grade or less grade	29503317 (11.87)	28265252 (11.96)	1238065 (10.19)		29502412 (11.87)	29138537 (11.89)	363876 (10.72)		29503317 (11.87)	29242336 (11.83)	260981 (18.67)	
High School diploma/GED	60346209 (24.28)	57312348 (24.25)	3033861 (24.96)		60346209 (24.28)	59384126 (24.22)	962083 (28.33)		60337915 (24.28)	59919559 (24.25)	418356 (29.93)	
Some college credit but no degree	53116557 (21.37)	49507555 (20.94)	3609002 (29.7)		53116557 (21.37)	52072535 (21.24)	1044022 (30.74)		53114951 (21.37)	52694554 (21.32)	420397 (30.08)	
Associate's degree	23261488 (9.36)	21960638 (9.29)	1300850 (10.7)		23261488 (9.36)	22850795 (9.32)	410693 (12.09)		23261488 (9.36)	23147224 (9.37)	114264 (8.18)	
College graduate or higher	82311787 (33.12)	79341065 (33.56)	2970722 (24.45)		82311787 (33.12)	81696580 (33.33)	615207 (18.12)		82298821 (33.12)	82115267 (33.23)	183554 (13.13)	
Employment status:				<0.001				<0.001				<0.001
Employed full time	123456806 (49.67)	117882860 (49.87)	5573946 (45.87)		123456806 (49.67)	122099156 (49.81)	1357650 (39.98)		123438897 (49.67)	122929270 (49.74)	509627 (36.47)	
Employed part time	32312251 (13.0)	30149676 (12.75)	2162575 (17.80)		32312251 (13.0)	31618021 (12.9)	694230 (20.44)		32310646 (13.0)	32052178 (12.97)	258468 (18.49)	
Unemployed	9790298 (3.94)	8789427 (3.72)	1000870 (8.24)		9790298 (3.94)	9400465 (3.84)	389832 (11.48)		9786946 (3.94)	9527987 (3.86)	258960 (18.53)	
Other (including not in labor force)	82980004 (33.39)	79564895 (33.66)	3415109 (28.10)		82979100 (33.39)	82024931 (33.46)	954169 (28.1)		82980004 (33.39)	82609507 (33.43)	370497 (26.51)	
Total family income:				<0.001				<0.001				<0.001
Less than $20,000	36453938 (14.67)	33625163 (14.22)	2828774 (23.28)		36453938 (14.67)	35462180 (14.47)	991757 (29.2)		36452332 (14.67)	35989730 (14.56)	462603 (33.10)	
$20,000–$49,999	70632874 (28.42)	66771778 (28.25)	3861095 (31.77)		70631969 (28.42)	69595532 (28.39)	1036437 (30.52)		70632874 (28.42)	70182791 (28.4)	450083 (32.20)	
$50,000–$74,999	39622927 (15.94)	37930226 (16.05)	1692701 (13.93)		39622927 (15.94)	39151750 (15.97)	471177 (13.87)		39609960 (15.94)	39423429 (15.95)	186531 (13.35)	
$75,000 or More	101829621 (40.97)	98059691 (41.48)	3769929 (31.02)		101829621 (40.97)	100933112 (41.17)	896509 (26.4)		101821327 (40.97)	101522991 (41.08)	298336 (21.35)	
Mental health disorder symptoms												
Past year alcohol use dependence:				<0.001				<0.001				<0.001
No	240727858 (96.86)	230048482 (97.32)	10679376 (87.88)		240726953 (96.86)	237821200 (97.01)	2905752 (85.57)		240704992 (96.86)	239519758 (96.92)	1185235 (84.81)	
Yes	7811501 (3.14)	6338376 (2.68)	1473125 (12.12)		7811501 (3.14)	7321373 (2.99)	490129 (14.43)		7811501 (3.14)	7599184 (3.08)	212318 (15.19)	
Past year illicit drug use dependence:				<0.001				<0.001				<0.001
No	243110203 (97.82)	232316012 (98.28)	10794191 (88.82)		243109298 (97.82)	240250766 (98.00)	2858533 (84.18)		243087338 (97.82)	241941772 (97.90)	1145566 (81.97)	
Yes	5429155 (2.18)	4070847 (1.72)	1358309 (11.18)		5429155 (2.18)	4891807 (2.00)	537348 (15.82)		5429155 (2.18)	5177169 (2.10)	251987 (18.03)	
Past year MDE symptoms:				<0.001				<0.001				<0.001
No	227491583 (92.11)	221645682 (94.35)	5845901 (48.4)		227490678 (92.11)	226346398 (92.91)	1144280 (33.97)		227478617 (92.11)	226914419 (92.40)	564198 (40.71)	
Yes	19492897 (7.89)	13259460 (5.65)	6233437 (51.6)		19492897 (7.89)	17268514 (7.09)	2224383 (66.03)		19482998 (7.89)	18661429 (7.60)	821568 (59.29)	

Note: Data Source: National Survey on Drug Use and Health (NSDUH) 2019; Statistical significance at p < 0.05; All p-values are based on chi-square tests for the categorical variables; MDE = Major depressive episode.

**Table 2.** Weighted multivariate logistic regression analyses of demographic characteristics, substance use, and mental health disorder symptoms associated with suicidality among US adults in 2019.

	Model 1		Model 2		Model 3	
	Suicidal Thoughts		Suicidal Plans		Suicidal Attempts	
	AOR	95% CI	AOR	95% CI	AOR	95% CI
Age:						
18–25	Ref		Ref		Ref	
26–34	0.67	(0.57, 0.78)	0.72	(0.55, 0.96)	0.71	(0.48, 1.03)
35–49	0.52	(0.45, 0.60)	0.64	(0.49, 0.83)	0.57	(0.40, 0.80)
50–64	0.38	(0.30, 0.49)	0.39	(0.26, 0.59)	0.23	(0.10, 0.54)
65 or Older	0.32	(0.23, 0.45)	0.33	(0.17, 0.68)	0.28	(0.09, 0.94)
Gender:						
Female	Ref				Ref	
Male	1.07	(0.94, 1.22)	1.00	(0.79, 1.27)	0.85	(0.59, 1.23)
Sexual identity:						
Heterosexual	Ref		Ref		Ref	
Lesbian or Gay	1.62	(1.23, 2.15)	2.91	(1.93, 4.39)	2.43	(1.24, 4.76)
Bisexual	2.15	(1.78, 2.61)	2.38	(1.86, 3.04)	2.12	(1.54, 2.90)
Race/Ethnicity:						
Non-Hispanic White	Ref				Ref	
Non-Hispanic Black/African American	0.74	(0.61, 0.90)			1.52	(1.11, 2.08)
Hispanic	0.94	(0.77, 1.14)			1.06	(0.67, 1.67)
Other race	0.82	(0.63, 1.06)			1.27	(0.82, 1.96)
Level of education completed:						
College graduate or higher	Ref		Ref		Ref	
Twelfth grade or less grade	1.04	(0.78, 1.39)	1.38	(0.84, 2.26)	2.96	(1.53, 5.71)
High School diploma/GED	1.15	(0.93, 1.42)	1.65	(1.16, 2.35)	2.15	(1.25, 3.69)
Some college credit but no degree	1.22	(1.01, 1.49)	1.46	(1.09, 1.96)	1.89	(1.07, 3.32)
Associate's degree	1.33	(1.03, 1.70)	1.88	(1.26, 2.80)	1.69	(0.81, 3.56)
Employment status:						
Employed full time	Ref		Ref		Ref	
Employed part time	1.16	(0.97, 1.39)	1.37	(1.04, 1.80)	1.19	(0.76, 1.88)
Unemployed	1.54	(1.21, 1.97)	2.05	(1.40, 2.99)	2.89	(1.79, 4.66)
Other (including not in labor force)	1.03	(0.87, 1.21)	1.12	(0.91, 1.38)	1.06	(0.74, 1.53)
Total family income:						
Less than $20,000	Ref		Ref		Ref	
$20,000–$49,999	0.85	(0.71, 1.03)	0.73	(0.55, 0.96)	0.75	(0.50, 1.11)
$50,000–$74,999	0.69	(0.57, 0.82)	0.68	(0.48, 0.94)	0.70	(0.44, 1.12)
$75,000 or More	0.69	(0.56, 0.85)	0.63	(0.47, 0.85)	0.57	(0.35, 0.92)
Mental health disorder symptoms						
Past year alcohol use dependence:						
No	Ref		Ref		Ref	
Yes	2.36	(1.88, 2.96)	2.00	(1.44, 2.77)	2.08	(1.34, 3.23)
Past year illicit drug use dependence:						
No	Ref		Ref		Ref	
Yes	2.03	(1.54, 2.69)	1.97	(1.42, 2.72)	2.14	(1.47, 3.13)
Past year MDE symptoms:						
No	Ref		Ref		Ref	
Yes	12.19	(10.21, 14.54)	15.42	(12.20, 19.50)	9.95	(6.35, 15.58)

Note: AOR = Adjusted odds ratio; 95% CI = 95% confidence interval; Statistical significance at p < 0.05; Ref = Reference group; MDE = Major depressive episode.

### 3.2. Multivariate logistic regression and margins analyses

Table 2 presents the results of the weighted multivariate logistic regression analysis.

Model 1: Suicidal thoughts. Compared to the population aged 18–25 years, those aged 26–34, 35–49, 50–64, and 65+ years had lower odds of suicidal thoughts in the past year. The populations that self-identified as lesbians, gays, or bisexuals had higher odds of suicidal thoughts compared to the heterosexual population. Relative to non-Hispanic Whites, non-Hispanic Blacks/African Americans were less likely to experience suicidal thoughts, whereas no statistically significant differences exist in experiencing suicidal thoughts between non-Hispanic Whites and Hispanics or non-Hispanic other racial/ethnic groups. Those who had some college credit but no degree or had an associate's degree had higher odds of suicidal thoughts compared to those who had a college graduate or higher degree. Suicidal thoughts were significantly higher for unemployed individuals (AOR = 1.54, 95% CI = 1.21, 1.97) relative to full-time employed individuals. The population that reported total family incomes of $50,000–$74,999 and $75,000 or more had lower odds of suicidal thoughts compared to those who reported total family income of less than $20,000. Experiencing suicidal thoughts was significantly higher for individuals with alcohol use dependence (AOR = 2.36, 95% CI = 1.88, 2.96) versus no alcohol use dependence; illicit drug use dependence (AOR = 2.03, 95% CI = 1.54, 2.69) versus no illicit drug use dependence; and MDE symptoms (AOR = 12.19, 95% CI = 10.21, 14.54) versus no MDE symptoms.

**Figure 1.**
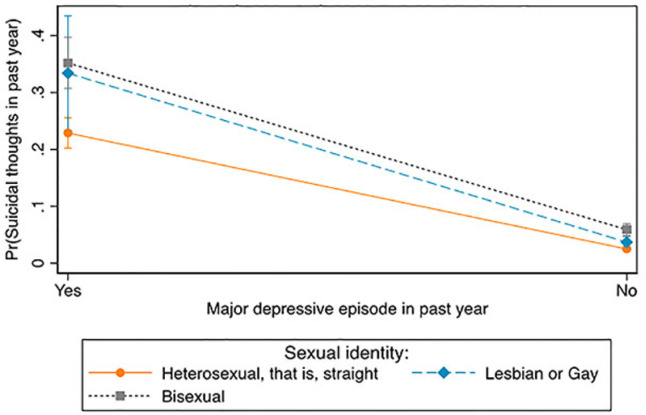
Differences in suicidal thoughts between and within major depressive episode and sexual identity.

Figure 1 shows between and within-group differences in suicidal thoughts by MDE symptoms and sexual identity. Those who identified as bisexuals and had experienced MDE symptoms (35%) had the highest probability of having suicidal thoughts compared to lesbians or gays (33%) and heterosexuals (23%) with MDE symptoms. Within groups, bisexuals who experienced MDE symptoms had a higher probability of having suicidal thoughts than bisexuals who had not experienced MDE symptoms (6%). Lesbians or gays who had experienced MDE symptoms had a higher probability of having suicidal thoughts than lesbians or gays who had not experienced MDE symptoms (4%). The probability of having suicidal thoughts was higher for heterosexuals who had experienced MDE symptoms than heterosexuals who did not experience MDE symptoms (3%).

Model 2: Suicidal plans. Individuals aged 26–64 or 65+ years had lower odds of suicidal plans in the past year relative to those aged 18–25 years. The odds of experiencing suicidal plans were significantly higher for individuals who self-identified as lesbians, gays, or bisexuals versus those who self-identified as heterosexuals. The population that had a High School diploma/GED, some college credit but no degree, or associate's degree had higher odds of suicidal plans compared to those who had a college graduate or higher degree. Those who were employed part-time or unemployed had higher odds of suicidal plans relative to full-time employed people. Suicidal plans were significantly lower for people who had total family incomes of $20,000–$49,999, $50,000–$74,999, and $75,000 or more compared to those who had less than $20,000. Compared to those who had no experience with alcohol use dependence, those who had experience with alcohol use dependence had 2.0-fold higher odds of suicidal plans (AOR = 2.00, 95% CI = 1.44, 2.77). Individuals who had experienced illicit drug use dependence, compared to those who did not, had 2.0-fold higher odds of suicidal plans (AOR = 1.97, 95% CI = 1.42, 2.72). Compared to people who had not experienced MDE symptoms, those who had experienced MDE symptoms had 15.4-fold higher odds of suicidal plans (AOR = 15.42, 95% CI = 12.20, 19.50).

**Figure 2.**
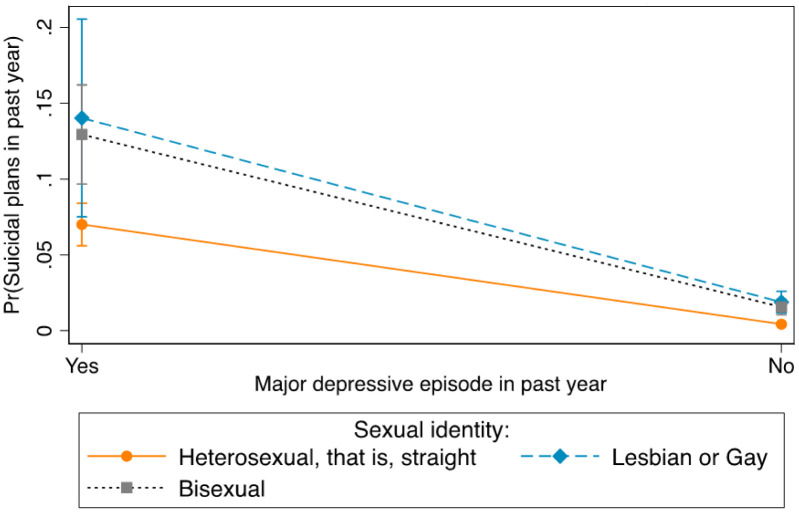
Differences in suicidal plans between and within major depressive episode and sexual identity.

Figure 2 shows differences in suicidal plans between and within MDE symptoms and sexual identity. Between groups, the probability of having suicidal plans was higher for lesbians or gays who had experienced MDE symptoms (14%) than for bisexuals (13%) and heterosexuals (7%) with MDE symptoms. Within groups, lesbians or gays who had experienced MDE symptoms had a higher probability of having suicidal plans than lesbians or gays who had not experienced MDE symptoms (2%). The probability of having suicidal plans was higher for bisexuals who had experienced MDE symptoms than for bisexuals who had not experienced MDE symptoms (2%). Heterosexuals with MDE symptoms had a higher probability of having suicidal plans than heterosexuals who did not experience MDE symptoms (0.4%).

Model 3: Suicidal attempts. Compared to the population aged 18–25 years, those aged 35–49, 50–64, and 65+ years were significantly less likely to experience suicidal attempts in the past year. The population that self-identified as lesbians or gays (AOR = 2.43, 95% CI = 1.24, 4.76) and bisexuals (AOR = 2.12, 95% CI = 1.54, 2.90) had higher odds of suicidal attempts compared to heterosexuals. Non-Hispanic Blacks/African Americans, compared to non-Hispanic Whites, were more likely to experience suicidal attempts, while no differences exist in suicidal attempts between Hispanics and non-Hispanic other racial groups versus non-Hispanic Whites. Compared to those who had a college or higher degree, those who had completed twelfth grade or less grade, High School diploma/GED, and some college credit but no degree had higher odds of suicidal attempts. The unemployed population, compared to the full-time employed population, had higher odds of suicidal attempts (AOR = 2.89, 95% CI = 1.79, 4.66). Those who had a total family income of $75,000 or more had lower odds of suicidal attempts (AOR = 0.57, 95% CI = 0.35, 0.92) compared to those who had less than $20,000. Experiencing suicidal attempts is significantly associated with alcohol use dependence (AOR = 2.08, 95% CI = 1.34, 3.23) relative to no alcohol use dependence. The population that had an experience with illicit drug use dependence had higher odds of suicidal attempts (AOR = 2.14, 95% CI = 1.47, 3.13) compared to those who did not experience illicit drug use dependence. Compared to those who did not have experience with MDE symptoms, those who had experience with MDE symptoms had higher odds of suicidal attempts (AOR = 9.95, 95% CI = 6.35, 15.58).

**Figure 3.**
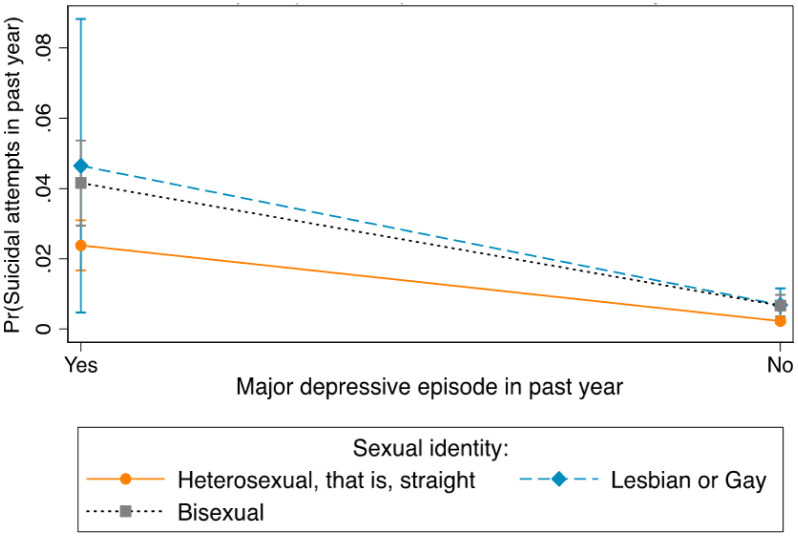
Differences in suicidal attempts between and within major depressive episode and sexual identity.

The differences in suicidal attempts between and within MDE symptoms and sexual identity groups were presented in Figure 3. Lesbians or gays who had experienced MDE symptoms (5%) had a higher probability of experiencing suicidal attempts compared to bisexuals (4%) and heterosexuals (2%) with MDE symptoms. The probability of experiencing suicidal attempts was higher for lesbians or gays who had experienced MDE symptoms than lesbians or gays who had not experienced MDE symptoms (0.7%). Bisexuals with MDE symptoms had a higher probability of experiencing suicidal attempts than bisexuals with MDE symptoms (0.7%). The probability of having suicidal attempts was higher for heterosexuals with MDE symptoms than for heterosexuals with no MDE symptoms (0.2%).

## 4. Discussion and conclusions

Our study found an association between the three suicidal domains and mental health disorder symptoms as well as sociodemographic characteristics, which offer several contributions to the literature. People with a past-year history of alcohol and illicit drug use dependence were twice more likely to experience suicidal thoughts or make an attempt at committing suicide. Our finding is consistent with other studies that showed an association between alcohol and drug use and suicidal ideation [32],[33]. Thus, people with a history of illicit drug use dependence and alcohol use dependence in the general population have a higher risk of suicidality [33]–[36]. This risk behavior may be due to mental health disorder symptoms such as severe depression that increase the risk for suicidality and predict a higher probability of suicide behaviors among people with alcohol or drug use disorders [37],[38]. While the chemical components in stimulants and alcohol are known to have deleterious effects on the brain with resultant impairment of judgment, withdrawal further exacerbates a feeling of stress, dysphoria, anxiety, and impending doom [39]–[41]. This complex process results in psychological distress and the negative effect that makes a user more vulnerable to suicidal thoughts, plans, or an actual attempt at executing a suicide plan [39],[41]. Importantly, our findings further support the necessity for integration of substance use treatment into routine suicidality prevention and treatment and health care delivery.

We found that the likelihoods of experiencing suicidal thoughts, plans, and attempts were more likely to occur in people with MDE symptoms compared to those without MDE symptoms. These findings suggest that there may be additional person-related factors specific to those with mental health disorder symptoms that make them more likely of experiencing suicidality. While this finding is alarming, it also supports the current literature [11],[12],[42]. People with major depressive symptoms are prone to having psychotic symptoms accompanied by hallucinations and a feeling of hopelessness, all of which may tilt the individual towards suicidality [43]. Our study suggests that a substantial number of people reporting MDE symptoms end up eventually with an attempt to commit suicide. This may indicate a delay in access to mental health counseling services. This further highlights the urgent need for enhanced capacity for health care workers, screening for mental health disorders, and prompt linkage to counseling services.

The ability to identify populations that may experience suicidality independent of MDE and substance use disorder symptoms would be a critical strategy to modifying potential risks at a population health level. Our findings suggest that several sociodemographic characteristics were associated with the suicidal domains. Suicidal thoughts, plans, and attempts tend to be more likely among young adults aged 18–25 years. This transitional age bracket from adolescence to adulthood may be overwhelming and lonely as the young adult navigates a new path to independence. Also, young adults tend to be more vulnerable to social pressures and may lack coping mechanisms to deal with life challenges compared to older populations. However, we did not find any statistical differences in suicidality between males and females, suggesting that adult males and females are likely to have similar experiences of suicidality. While non-Hispanic Black people were less likely to report suicidal thoughts, they were more likely to attempt suicide. There is an inconsistency in the literature regarding the burden of suicidal thoughts and attempts among different racial-ethnic groups [44]. However, it is known that protective factors, including an individual's coping mechanism, presence of social support system, strong family ties, spirituality, and access to mental health counseling services, minimize the risk of suicidality [44]–[46]. Most of these factors tend to vary within and between different racial-ethnic groups, with racial/ethnic minorities disproportionately less likely to have access to these protective resources. Additionally, we found that being educated beyond some college degree or having an associate's degree, and being unemployed was associated with more likelihoods of suicidal thoughts and attempts, while higher total family income was protective. Contrary to our findings on education, a study found that educational attainment reduced the likelihood of suicidal attempts independent of mental health disorders or cognitive performance [47]. The differences in the association between suicidal attempts and educational attainment in our study and this previous study could be due to differences in age groups and measures of education. Our study sample included people aged at least 18 years and education was measured as a categorical variable, but the previous study included participants aged 15–34 years and education was measured as a continuous variable. Thus, people of different age groups have different characteristics and experiences, including educational attainment, which may disproportionately influence suicidality among them.

Our results also suggest that sexual minority groups (lesbians or gays and bisexuals) have over two-fold likelihood of having suicidal plans and attempts. Similarly, other studies have shown that sexual minority people have a higher lifetime prevalence of suicide attempts than heterosexuals [22],[48]. Findings from a systematic review and meta-analysis indicate that one in five sexual minority adults have attempted suicide [22]. Also, individuals who identify as sexual minorities compared to heterosexuals showed higher odds of MDE symptoms and suicidal thoughts, plans, and attempts. Whereas bisexuals showed a higher probability of suicidal thoughts than lesbians and gays, lesbians and gays showed a higher probability of suicidal plans and attempts than bisexuals. This may be due to reports of major depression, generalized anxiety disorders, comorbid diagnoses, and substance abuse/dependence among sexual minority individuals [49]. About one-third of people who identify as sexual minorities attempt suicide in their lifetime and meet the diagnostic criteria for a mental health disorder [49]. Furthermore, our study revealed that suicidal thoughts, plans, and attempts are heightened among lesbians or gays, and bisexuals who reported MDE symptoms compared to those without MDE symptoms. This is consistent with previous studies linking mental health disorders to suicidal behaviors, and may be related to the continued stigma and discrimination among sexual minority groups which disproportionately affects their mental health [20]–[22]. Additionally, sexual minority individuals frequently experience unique stressors such as homelessness, discrimination, stigma, physical and sexual abuse, and mental health disorder symptoms including depression all of which are significant risk factors for suicidal ideations and behaviors [20],[50]–[52]. This finding is relevant for targeted public health interventions to address suicidality among sexual minority groups. However, we did test for male/female differences by sexual orientation but these were not significant, indicating that the effects of sexual orientation are the same across gender identity. This is not surprising as our findings revealed that there were no differences in suicidality between males and females.

A strength of this study is that it utilizes a national dataset and DSM variables, hence a representative sample of the population with generalizable results. However, there are a few limitations. Given that this was a cross-sectional study, we cannot infer causality. As such, it is unclear whether the MDE symptoms caused alcohol and drug use disorders which eventually led to suicidality, or if other risk factors of suicidal behaviors led to MDE symptoms and alcohol and drug use disorders. Also, the study only focused on alcohol use dependence, illicit drug use dependence, and MDE symptoms in the past year. This inherent aspect of the survey impeded our ability to look at the dose-response association between alcohol or illicit drug dependence and suicidality. Moreover, we could not assess the association between length of MDE and suicidality. As a result, we cannot make any inferences regarding the length of exposure and the dose-response association. Additionally, the NSDUH did not capture the frequency of each of the suicidality domains, and therefore, we were unable to assess these frequencies. The study, however, provided evidence of the strong association between MDE symptoms, substance use disorders, and suicidality among sexual minority and heterosexual populations.

In conclusion, we found an association between mental health disorder symptoms, substance use disorder symptoms, and sociodemographic characteristics with suicidal thoughts, plans, and attempts. People with substance use dependence, MDE symptoms, young people, family income of less than $20,000, unemployed, less than college graduate or higher education, non-Hispanic Blacks, and sexual minorities had higher burdens of suicidality. MDE symptoms were associated with increased suicidality among sexual minority people, which implies that sexual identity moderated the association between MDE symptoms and suicidality. Also, the prevalence of the three domains of suicidality assessed was higher among people with a past-year history of MDE symptoms regardless of sexual identity. However, we found higher risks of suicidal thoughts, plans, and attempts among sexual minority groups (lesbians or gays and bisexuals). These findings underscore the need for tailored interventions and policies to enhance the provision of prompt mental health screening, diagnosis, and linkage to care for mental health services, particularly among the most vulnerable in the population. Additionally, our findings suggest the need to critically consider MDE symptoms and substance use dependence in suicidality treatment and prevention, especially among sexual minorities.
